# A new wave in the quiet revolution in contraceptive use in Nepal: the rise of emergency contraception

**DOI:** 10.1186/s12978-016-0155-7

**Published:** 2016-05-04

**Authors:** Shyam Thapa

**Affiliations:** Nepal Public Health Foundation, Kathmandu, Nepal; Pokhara University, Kaski District, Nepal

**Keywords:** Emergency contraception, Users' profile, Nepal

## Abstract

**Background:**

In Nepal, while the use of the emergency contraceptive pill (ECP) has been increasing rapidly in recent years, very little is known about the profile of ECP users. This study assesses the increasing role of ECP and the profile of ECP users in Nepal. Some policy and programmatic concerns are also addressed.

**Methods:**

Survey interviews were undertaken involving 185 women who visited five metropolitan medical shops located in Pokhara seeking to purchase ECP for their own use. Descriptive statistics were calculated for data analysis.

**Results:**

ECP is popular among young (<25 years old) and educated women. Also, nearly 70 % of the ECP users described their most recent sexual relationship as infrequent/casual. The overwhelming majority of users are aware that ECP is for emergency use only. Most ECP users are also aware of other options like condoms, the oral pill, and injectable contraceptives; and a considerable proportion of women using ECP had also used these methods before.

**Conclusion:**

ECP is filling an important and unique role in reducing unplanned or unwanted pregnancy, especially among young, educated women, and those with infrequent sexual relationships.

## Background

The emergency contraception pill (ECP) is the only postcoital contraceptive method to prevent pregnancy [1]. In many countries, ECP is available over-the-counter without a prescription, thus making access easy (www.emergencycontraception.org). As Coeytaux, Wells and Westley have noted, ECP "expands choice, not only because it provides a unique postcoital opportunity, but also because women can access it for themselves with minimal medical supervision …" [2].

In Nepal, ECP was first introduced by the social marketing program in 2004. Initially, sales of ECP experienced only a modest increase until mid-2009. However, from 2009 onward, ECP sales increased rapidly, and during the period from mid-2014 to mid-2015, stood at about 500,000 doses sold, implying 25,000 couple-years of protection [3]. These data do not include the sale of various other commercial (that is, non-social marketing) brands that are also available on the market. Depending on the brand, an ECP costs between Rs. 100 and Rs. 160 (an equivalent cost of about $1 to $1.60 in October 2014).

The social marketing program data suggest that ECP is quietly making inroads in Nepal. The recent and rapid rise in use could be related to both supply and demand aspects. As regards the former, various ECP brands are available over-the-counter, principally in medical shops (pharmacies/drug stores), throughout the country. On the demand generation side, ECP is freely advertised on television and through mass media in print, with some restrictions applied to specific brands. Further, unlike the contraceptive oral pill, which has never been very popular in Nepal [4], ECP is not associated with family planning programs conventionally targeted at married couples and primarily for fertility-limiting purposes. Further, as a result of globalization, it may be expected that sexual relationships are no longer limited to traditionally-defined marital unions. The age at marriage has been rising, and the percentage single has also increased over the years [5]; both these changes have most probably contributed to the emerging sexual revolution. The convenient, over-the-counter availability of ECP also provides an option for many women seeking to prevent an unintended--unplanned and unwanted--pregnancy without having to queue up in a clinic or potentially face social stigmatization.

While the rapid rise in the use of ECP is a welcome development towards minimizing unintended pregnancies, it is not free of concern. Given the free marketing and wide availability of ECP, some (including medical shop keepers) have begun to raise concerns as to ECP's potential misuse and overuse. Similarly, concerns have also been raised in policy and reproductive health program circles. More specifically, some have pointed out that ECP may be being used as a substitute for other contraceptive methods in Nepal. In recent years, this issue has been more poignant in dialogues and discussions, especially in light of the apparent stagnation of contraceptive prevalence in the country [6, 7]. The concern regarding the potential use of ECP as a substitute for contraceptive use also parallels concerns raised in response to the rising use of abortion in Nepal [8]. One of the ubiquitous advertisements for ECP, for example, stating "Easy way for a difficult situation" on public hoarding boards and in print media may be, it is alleged, misleading potential contraceptive users, and making them either abandon or consider not using a contraceptive method regularly. In addition, globally, there has also been concern in recent years that ECP, at the population level, is not as effective as it was once thought to be [9]. While there is concern in Nepal about "overuse," this concern is not supported in the literature. As the International Consortium of Emergency Contraception notes: "A large number of studies show, however, that women and adolescents with greater access to EC are not more likely to engage in unprotected intercourse, and are actually more likely to adopt an ongoing contraceptive method after EC use" [10].

The rapid rise in ECP use in Nepal begs several questions: who is using the product and what brands are preferred and most frequently used? How often it is being used? Are the ECP users aware of other contraceptives and have they used them previously? Among ECP users, are they aware of pregnancy tests or, eventually, medical abortion should ECP fail? The 2011 Nepal Demographic and Health Survey did not collect data on awareness or use of ECP [4]. Very little is known about the users of ECP in Nepal.

In view of the lack of any systematically available information on increasing ECP use in Nepal, we undertook a formative-type descriptive study which could provide preliminary data regarding some of the concerns discussed above. More specifically, the study sought to: (i) Assess the profile of ECP users and their awareness and use of other contraceptives, (ii) understand the reasons and context for use of ECP, and (iii) explore the users' marketing and product preferences. This paper presents the main results of the study.

## Methods

The ECP study was undertaken as a nested study, part of a larger study in Pokhara, the second largest metropolitan city in Nepal, with a population of about 500,000. The pharmacy/drug wholesalers in Pokhara estimated that about 15,000 ECP per month are currently used, and that the demand for ECP has been rising over recent years. Of the five brands generally available -- I pill®, E con®, Max 72®, E 72®, and Unwanted® -- E con® is the only socially-marketed ECP brand, and sells for Rs.100 (equivalent to about $1).

We selected five medical shops for intercepting females coming to purchase ECP for self-use. The shops were purposely selected based on the following criteria: (i) location in socio-economically diverse communities in the metropolis, (ii) having a relatively high number of clients, based on client flow in the past month, (iii) space available to conduct interviews in private, (iv) and willingness of the shop operator to allow interviews to take place and to facilitate the interview process, subject to consent of the customers/clients. Three shops were located in the central area of the city (one was relatively large with a mini-clinic and two were based near hospitals), the fourth was located in a periphery area of the city, and the fifth shop was located adjacent to a sizeable poor neighborhood.

The eligible respondents for the survey were females who came to the shop seeking ECP for themselves. We excluded male customers seeking to purchase medication and any females who reported that they were there to buy ECP on behalf of their friends. One interviewer was stationed in each shop. In only one shop the interviewer stayed through the late afternoon; in other cases interviews were generally completed around 5 PM.

The shop operators were required to keep a daily record of the total number of males and females who came to the shop to purchase ECP. We aimed to randomly intercept 2–4 ECP customers per day in each shop and ask them to participate in the survey, subject to their consent. In the absence of any particular hypothesis to test, we arbitrarily aimed to obtain, over a period of about two weeks, a sample of around 175 respondents from the participating shops.

The survey instrument was mostly structured. It included about 60 questions and was designed to take 20–25 min per interview. The instrument included questions relating to the following aspects: client's background; awareness and use of various brands of ECP; awareness and use of a pregnancy test, ultrasound, and medical abortion; and awareness of, ever use, and most recent use of contraceptives. The responses to open-ended questions were coded later. In order to guarantee privacy and confidentiality, respondents were not asked their names or addresses.

During the 15-day study period (which excluded holidays), a total of 371 female and 281 male customers seeking to buy ECP visited the five shops. There were, thus, 1.3 female buyers for each male buyer. Of the 371 female buyers, 185 (49.9 %) were successfully interviewed. In shops with a low-frequency of customers, all eligible females were approached for an interview, while in the shops with medium and high customer frequency, every second or third eligible customer was approached for an interview. Of the total eligible respondents approached, about 20 % declined to be interviewed or did not complete the interview (on account of time or unwillingness to share part of the requested information). The non-response rate was considerably higher for younger females than for relatively older females. The number of respondents successfully interviewed in the five shops ranged from 1.9 to 2.6, with an overall average of 2.4 respondents per day. The data analyzed are based on a total of 185 respondents. Given the nature of the study, only descriptive statistics are presented. The term current buyers and current users are used interchangeably.

## Results

Of the five ECP brands currently available on the market, I pill® and E con® are the two that are the most commonly known and the most commonly utilized by the current users of ECP (Table 1). Just over 78 % of ECP users had heard of these brands. I pill was previously used by 50 % of the current ECP buyers followed by E con (45 %). Max 72 was used by only 17 %. Additionally, nearly 90 % of current buyers have used at least one brand previously. Among those who have used ECP in the past, the median weeks ago used was 10, and median times used was 3 (Table 2). Similarly, 20 hours was the median time elapsed since the most recent sexual activity, and the median time of last menstrual period was 17 days ago.

**Table 1:** Percentage aware of and ever used emergency contraceptive pill (ECP) by current users of ECP, Pokhara, 2014

	Ever heard		Ever used	
Brand®	Percentage	N	Percentage	N
I pill	78.4	145/185	50.3	93/185
E con	78.4	145/185	44.9	83/185
Max 72	36.2	67/185	16.8	31/185
E 72	34.6	64/185	9.7	18/185
Unwanted	27.6	51/185	7.0	13/185
Brand unknown	2.2	4/185	2.2	4/185
Any	97.8	181/185	88.1	163/185

**Table 2:** Prior use of emergency contraceptive pill (ECP) and timing of last sexual activity among current users of emergency contraception, Pokhara, 2014

	Range	Median (Mean)	N
Prior ECP use (weeks ago)	1–52	10.0 (14.1 ± 12.8)	163
Number of times ECP used in the past	1–25	3.0 (4.3 ± 4.0)	163
Timing of the most recent sexual activity (hours earlier)	2–80	20.0 (23.4 ± 16.2)	182
Timing of last menstrual period (days ago)	2–40	17.0 (17.3 ± 6.6)	182

20 respondents did not use the ECP in the past. Three cases were missing for the last two variables

Nearly half (47 %) of the ECP users were under 25 years of age (Table 3). Just over 70 % had completed 10^th^ grade or higher. Thirty percent were single. Of those married, 17.2 % reported their spouse as being away from home. About 50 % did not have a living child. The lifetime desired median number of children among ECP users surveyed was 2 (not shown in the table).

**Table 3:** Profile of the current users of emergency contraceptive pill (ECP), Pokhara, 2014

Variable	%	N
Age group		
<25	47.0	87
25–34	45.4	84
35 or older	7.6	14
Total	100.0	185
Educational attainment		
Less than primary	5.4	10
Primary	9.2	17
Secondary	15.1	28
High school (10^th^ grade)	24.9	46
Intermediate (10 + 2)	33.0	61
Bachelor’s or above	12.4	23
Total	100.0	185
Current marital status		
Single	29.7	55
Married	69.2	128
Divorced/Separated	1.1	2
Total	100.0	185
If married, whether husband is away		
Yes	17.2	22
No	82.8	106
Total	100.0	128
Number of living children		
0	49.7	91
1	31.7	58
2 or more	18.6	34
Total	100.0	183^a^

^a^2 missing cases

The overwhelming majority of respondents (94 %) reported that their sexual relationship was consensual (Table 4). Just over 90 % reported that the person with whom they had the relationship was a well-acquainted person. About two in five reported that the sexual encounter was completely spontaneous (that is, unplanned and unanticipated). One-third of all respondents reported that the decision to use ECP was their own. Of all the users, about 70 % described their current sexual relationship as infrequent.

**Table 4:** Decision regarding use of emergency contraceptive pill (ECP) and nature of relationship with the person among current users of emergency contraception, Pokhara, 2014

Variable	Percentage	N
Percentage reporting that sexual relationship was consensual	94.0	172/183
Percentage who reported that the sexual relationship occurred with a well-acquainted person	92.3	168/182
Percentage who reported that that sexual relationship was completely spontaneous (unplanned and unanticipated)	45.9	83/181
Percentage who reported that it was their own decision to use emergency contraception	32.8	60/183
Percentage who reported that they have infrequent/casual sexual relationship	69.7	124/178

Some categories do not add to 183 due to missing cases

All of the respondents were aware of other contraceptive methods including condoms, the pill, or injectables (Table 5). Further, 88 % had used a condom before and 40 % had used the oral pill. Injectables were used by about one-fourth of the respondents. About one in five women surveyed reported using either rhythm or withdrawal previously. Of the prior contraceptive method users, condoms were used by 61 % of women, followed by the pill (19 %), and lastly, injectable and withdrawal methods were used by 7 % each (not shown in a table). Table 6 shows the time when the last contraceptive method was used -- the median times for condom and pill usage were 2 and 4 weeks, respectively; and for withdrawal or rhythm methods, it was 1 week ago (the methods were combined in the analysis due to the small numbers of cases).

**Table 5:** Percentage aware and ever used contraceptive methods prior to the current use of emergency contraceptive pill (ECP), Pokhara, 2014

	Ever heard		Ever used	
Method	Percentage	N	Percentage	N
Condom	100.0	185/185	88.1	163/185
Oral pill	93.0	172/185	40.0	74/185
Injectable	92.4	171/185	24.3	45/185
IUD	28.6	53/185	0	0/185
Implants	35.7	66/185	0.5	1/185
Withdrawal	48.1	89/185	24.9	46/185
Rhythm	43.8	81/185	20.5	38/185
Condom, pills or injectable	100.0	185/185	94.6	175/185
Any of seven methods	100.0	185/185	96.2	178/185

**Table 6:** Time when last contraceptive method was used (in weeks), Pokhara, 2014

Method	Range	Median (Mean)	N
Condom	0.14–52	2.0 (3.6 ± 6.1)	108
Oral pill	0.14–64	4.0 (10.3 ± 14.2)	35
Injectable	0.71–104	16.0 (22.6 ± 24.8)	13
Withdrawal or Rhythm	0.14–5	1.0 (1.5 ± 1.5)	17

When asked (with an open-ended question) about the main reason for not using a condom at the time of their last sexual relationship, 26 % of the respondents reported it was a hassle and a nuisance to put on a condom before sex – and trying to do so inhibited spontaneity. Another one fourth of all respondents said that they had either run out of condoms or condoms were not readily available. Further, 19 % said either they themselves or their sexual partner preferred a natural feel and elected not to use a condom for a more intimate feeling (not shown in a table).

Nearly all (96 %) of those interviewed were aware that ECP is meant only for emergency purposes (not shown in a table). Approximately half wished to obtain additional information regarding ECP, while the rest of the respondents said they simply wanted to get ECP from the medical shop. About 57 % of respondents preferred ECP in a one-tablet regimen. The majority (77 %) had no preference for any particular brand of ECP. Four in five ECP users said they also knew friends who had used ECP.

Among the main source of information for ECP use, friends were the most commonly mentioned source (70 %), followed by TV (30 %), health personnel (27 %) and sexual partners (18 %). (These responses, not shown in a table, are based on multiple responses).

The majority (73.5 %) of the ECP users had used a pregnancy test in the past (Table 7). About two-thirds of those who were aware of ultrasound availability had used it sometime in the past. About 84 % had heard of medical abortion. Among those who had heard of it, one in five had used medical abortion in the past. Just over 50 % of ECP users intended to use medical abortion should ECP fail.

**Table 7:** Awareness of, use, and intent to use medical abortion, pregnancy test, ultrasound among current users of emergency contraceptive pill (ECP), Pokhara, 2014

Heard and Used	Percentage	N
Ever used pregnancy test kit	73.5	136/185
Ever heard of ultrasound	78.4	145/185
Ever done ultrasound^a^	65.5	95/145
Intends to use ultrasound in the future^a^	31.0	45/145
Ever heard of medical abortion	84.3	156/185
Ever used medical abortion^a^	19.9	31/156
Intends to use medical abortion if ECP does not work^a^	53.2	83/156

^a^Among those aware of given technology/product

We also inquired (through an open-ended question) as to why respondents wanted to rush to use ECP rather than first having a pregnancy confirmed and then making a decision. Just over half (57 %) of women said they were certain that they did not wish to become pregnant, but were fearful that they might be pregnant (not shown in a table). Hence, rather than taking a chance and resorting to a potentially more complicated and more expensive treatment later on, they wished to eliminate the possibility of a pregnancy at the earliest possible opportunity. About another one-fourth of the female respondents reported that they didn't want to take any chance later on as they were either unmarried or divorced (hence, potentially having to face family and social disgrace).

## Discussion

ECP is a relatively new addition to Nepal’s contraceptive repertoire -- and its use has risen particularly in the last 4–5 years. The data analyzed here shed light on the ways ECP is making inroads among contraceptive users, and seems to be a popular option, particularly among single and educated women in Nepal. The proportion of single women may have been underestimated in the data, as a disproportionate number of those not consenting to be interviewed were relatively young and suspected to be single. Additionally, based on our conversations with some of the medical shop operators at the time of the survey questionnaire design, we suspected that ECP may be making some headway among young, educated women -- and those with infrequent sexual relations. The data presented here provide some evidence to this emerging phenomenon, although the sample size is inadequate to fully test this hypothesis.

Among the various brands, I pill® and E con® are most popular, although the former is about 50 % more expensive than the latter. These two brands are popular on different grounds: I pill® because it is advertised heavily on Indian electronic media which is both available and accessible throughout much of Nepal, and E con® because it is cheaper and also advertised on television channels in Nepal. (Only the socially-marketed product—E con®—is permitted to be advertised on television). What might have happened to the relative popularity and, hence, market share, of these two products if I pill® (or for that matter other commercial brands) could be advertised through the national TV channels in Nepal, or if the prices were relatively similar, is unknown. Further, it has been established that the commercial brands offer heavy discounts (as much as 300 %), whereas the social-marketing brand has a less than 20 % margin. Clearly, the various brands are operating with different mechanisms, and the socially-marketed and commercial brands are not operating on the same playing field.

The increasing availability and use of ECP may also be one reason for the continuous decline in fertility, which is not completely explained by looking at the relationship between contraceptive use and fertility among married women [11, 12]. The increasing use of the ECP may also be one reason why, despite availability and efforts to cater to younger women, the proportion of young women using abortion remains relatively small [8].

TV remains an important source of information regarding the range of ECP products. The overwhelming majority of the users of ECP know that it is for emergency use only. Furthermore, all of the ECP users interviewed reported having used condoms before, and a significant proportion had also used the oral contraceptive pill and injectables. The majority of women described their recent sexual relationship as being infrequent and casual – and the fact that nearly one-third of the ECP users also reported being unmarried also signals that a sexual revolution is underway in Nepal. This is an entirely different situation than it was some 15 years ago [13]. Among the married women, that nearly one out of five women reported husbands being away from home also indicates temporary (voluntary) spousal separation as being another contributory factor influencing ECP use. Further, given the increasing population mobility (often male selected) within the country [14] and outside Nepal mostly for employment [15] in recent years, spousal separation is likely to remain an important factor for driving demand and continued use of ECP in Nepal.

The findings refute the often-made allegation in policy and reproductive health circles in Nepal that with wider availability and easy access ECP is being abused or misused. To the contrary, ECP may have found a niche, and it could be what has been described as a "bridge" [2] to the eventual use of contraception as women (and their partners) are in more regular and frequent relationships. Further, that the majority of the current ECP users would consider using medical abortion, should ECP fail to work, suggests that the ECP users utilize ECP as a convenient and immediate step. Based on the ECP users' awareness of the other various available contraceptive methods, as well as their use of other contraceptive methods, and the nature of their sexual relationships, it also appears groundless to assert that ECP is being used as a substitute for regular contraceptive use among the majority of users. At the same time, it seems important to design and provide more informational materials on ECP, as many users would like to get additional information. Additionally, in terms of cost, one dose of ECP costs $1 to $1.60, and a pregnancy test kit costs about $0.50. In contrast, medical abortion costs about $7.25. Clearly, for those who are aware of the cost of these products, ECP is the cheaper option. Further, as the present data suggest, some women do not want to wait until a pregnancy is confirmed, but rather would prefer to have peace of mind right away by taking ECP to eliminate any possibility of a pregnancy. Overall, based on the sexual and reproductive behavior of the women surveyed, the study suggests the emergence and increasing use of ECP is serving in the continuum of contraceptive option available in Nepal. Women afraid of an unwanted pregnancy, or suspecting an unintended pregnancy, have the option to use ECP long before resorting to a pregnancy test or ultrasound, which could eventually lead them to seek an abortion (most probably medical abortion). Following an abortion, many women may choose to use contraception. For many women, ECP seems, therefore, to serve as an early option in preventing unintended pregnancy.

### Limitation

This is a descriptive study; as such no specific hypothesis was developed and tested. Also, the study was conducted in purposively selected, high-cliental medical shops in a single metropolis in Nepal. The profile of the ECP users and the market share of the various brands of ECP may be different in other geographic locations in the country. The sample was not based on probability, but rather it represented only those who visited the shops during a specific window of time, determined on the basis of financial and logistical feasibility. Although men represented about 50 % of the ECP buyers, only women were interviewed, and it is unknown whether the characteristics of ECP users in which the men purchased the product were similar to those in which women themselves made the purchase. These limitations notwithstanding, this study provides some hitherto unavailable evidence and presents insights upon which future studies could be developed.

## Conclusion

Our study based in urban medical shops in Nepal found that ECP is popular particularly among young (<25) and educated women. The overwhelming majority of users are aware ECP is for emergency only. Nearly two-thirds of the ECP users described their sexual relationship as infrequent/casual. Most ECP users are aware of condoms, the oral contraceptive pill, and injectable contraceptives, and a considerable proportion of them have used these methods before. ECP seems to be filling an important niche in reducing unplanned or unwanted pregnancies. In light of the increasing use of the method, it would also be important to investigate real lifetime effectiveness with the ECP used. In future surveys (such as the Nepal Demographic and Health Surveys) it will be important to collect information on ECP use by including it in the repertoire for the contraceptive use module.

## References

[1] Stewart F, Trussell J, Van Look PFA, Hatcher RA, Trussell J, Nelson AL (2007). Emergency contraception. Contraceptive Technology.

[2] Coeytaux F, Wells ES, Westley E (2009). Emergency contraception: have we come full circle?. Contraception.

[3] http://www.crs.np Accessed 12 November 2015.

[4] MoHP (Ministry of Health and Population Nepal), New ERA, and ICF International Inc (2012). Nepal Demographic and Health Survey 2011.

[5] Bajracharya G, Bhandari DR (2014). Nuptiality trends and differentials in Nepal. Central Bureau of Statistics. Population Monograph of Nepal. Vol. I.

[6] Sharma G (2011). Revitalization of family planning in Nepal: Background paper submitted to DFID.

[7] Ban B, Karki S, Shrestha A, Hodgin S (2012). Spousal separation and interpretation of contraceptive use and unmet need in rural Nepal. International Perspectives on Sexual and Reproductive Health.

[8] Thapa S, Neupane S, Basnett I, Read E (2012). Women having abortion in urban Nepal: 2005 and 2010 Compared. Kathmandu University Medical Journal.

[9] Raymond EG, Trussell J, Polis C (2007). Population effect of increased access to emergency contraceptive pills: A systematic review. Obstet Gynecol.

[10] International Consortium for Emergency Contraception (ICEC).www.cecinfo.org/ec-issues. Accessed 28 February 2016.

[11] Karki YB, Krishna R (2008). Factors responsible for the rapid decline of fertility in Nepal—An interpretation: Further analysis of the 2006 Nepal Demographic and Health Survey.

[12] Khanal MN, Shrestha DR, Panta PD, Mehata S (2013). Impact of male migration on contraceptive use, unmet need and fertility in Nepal. Further analysis of the 2011 Nepal Demographic and Health Survey.

[13] Xenos P (2006). Delayed Asian transitions to adulthood: A perspective from national youth surveys. Asian Population Studies.

[14] Suwal BR (2014). Internal migration in Nepal. Central Bureau of Statistics. Population Monograph of Nepal. Vol. I.

[15] World Bank (2011). Large-scale migration and remittance in Nepal: Issues, challenges, and opportunities. Poverty Reduction and Economic Management Sector Unit, South Asia Region. Report No. 55390-NP.

